# Stimulation of cell proliferation in the subventricular zone by synthetic murine pheromones

**DOI:** 10.3389/fnbeh.2013.00101

**Published:** 2013-08-06

**Authors:** Sachiko Koyama, Helena A. Soini, John Foley, Milos V. Novotny, Cary Lai

**Affiliations:** ^1^The Linda and Jack Gill Center for Neuroscience and Department of Psychological and Brain Sciences, Indiana UniversityBloomington, IN, USA; ^2^Institute for Pheromone Research and Department of Chemistry, Indiana UniversityBloomington, IN, USA; ^3^Medical Sciences, Indiana University School of MedicineBloomington, IN, USA; ^4^Department of Dermatology, Indiana University School of MedicineIndianapolis, IN, USA

**Keywords:** pheromones, enhanced cell proliferation, subventricular zone, male, female, mice

## Abstract

Adult neurogenesis in female mice is known to be enhanced by exposure to soiled bedding from males, although the identity of the relevant chemosignals has remained unknown. Here we show that the previously recognized male murine pheromones, the farnesenes and 2-*sec*-butyl-4,5-dihydrothiazole (SBT), strongly increase cell proliferation in the subventricular zone (SVZ) of adult female mice, but not younger female mice. In addition, we found that a unique female murine pheromone, 2,5-dimethylpyrazine, facilitates similar changes in males. SBT stimulated cell proliferation in the SVZ of only adult females and not in young adult or pre- and post-puberty females. Our study suggests that pheromonal communication between males and females is enhancing reproductive success by controlling the estrous cycle and by promoting cell proliferation in a reciprocal manner.

## Introduction

Chemical communication through pheromones is widespread in nature. The classical definition of pheromonal function recognizes the signaling effects that influence behaviors and the primer effects that mediate physiological changes in conspecifics (Wilson and Bossert, 1963). The early examples of specific olfactory messengers in mammals are estrus suppression (Lee-Boot effect) (Lee and van der Boot, 1955, 1956) and estrus synchrony (Whitten effect) (Whitten, 1956, 1958), first observed in the house mouse (*Mus domesticus*) (reviewed in Koyama, 2004). The identification of these messengers has been based on detection in animal fluids, and the demonstration that synthetic versions of these molecules were capable of eliciting the appropriate biological effects (Jemiolo et al., 1986; Novotny et al., 1986; Ma et al., 1998, 1999; Koyama, 2004). More recently, a novel olfactory effect was described, in which soiled bedding from male mice induced an increase in neurogenesis within the subventricular zone (SVZ) (Mak et al., 2007; Larsen et al., 2008; Oboti et al., 2009) and dentate gyrus (Mak et al., 2007) of recipient adult females. However, the identity of the relevant olfactants has remained unknown and these studies focused on females. There have been no reports concerning olfactory effects on neurogenesis or cell proliferation in the brains of males.

In the studies on the impact of male-soiled bedding on neurogenesis in females, the soiled bedding from dominant males induced higher levels of neurogenesis than the bedding from subordinate males (Mak et al., 2007). In our previous studies on olfactory communication, the compounds dehydro-exo-brevicomin (DHB), 2-*sec*-butyl-4,5-dihydrothiazole (SBT), E,E-α-farnesene and E-β-farnesene (Jemiolo et al., 1986; Ma et al., 1999) were shown to mediate the Whitten effect, i.e., the effect of inducing estrus in females (Koyama, 2004). These 3 compounds have also been detected at higher concentrations in the urine of dominant vs. subordinate males (Novotny et al., 1990). We thus hypothesized that these compounds may be excellent candidate chemosignals that stimulate neurogenesis in female mice.

Previous studies have focused on the influences of male-soiled bedding on neurogenesis in females. In earlier work, we showed that female-soiled bedding enhanced sperm density in males (Koyama and Kamimura, 2000). The influence was stronger (i) when males were exposed to the soiled bedding of females housed in groups than when they were exposed to the soiled bedding of the same number of females housed individually and also (ii) when the females were housed at higher density (unpublished data of SK). We have previously shown that 2,5-dimethylpyrazine (2,5-DMPZ) is secreted specifically by females that are kept in groups and it causes the suppression of their estrous cycles (Lee-Boot effect) (Lee and van der Boot, 1955, 1956; Novotny et al., 1986; Ma et al., 1998). This suppression is stronger when females are kept at higher density (Coppola and Vandenbergh, 1985). These studies showed that males are affected by female-soiled bedding. The studies also suggested that, if female-soiled bedding affects the males' reproductive condition, neurogenesis may also be influenced as gonadal hormones are known to be related to neurogenesis (Galea et al., 2006). As 2,5-DMPZ is known to be secreted at higher concentration by females housed in groups, and as group-housed females have a stronger impact on male sperm density, we hypothesized that this compound may affect neurogenesis in males.

## Materials and methods

### Animals

C57BL/6 mice (120 females and 53 males, which includes the odor donor mice) were utilized. Mice were bred in-house to ensure that they had no prior contact with adult male scent after they are weaned. All the mice were housed in individually ventilated isolation cages, connected to a tube providing filtered air from outside of the building, so there was no odor contamination between cages. Mice were used at 11–12 weeks of age to determine the influences of exposure to pheromones and soiled beddings on cell proliferation in the adult brain. The initial ages of the mice used to measure pheromone influence as a function of developmental stage were as follows: 24, 30, 34, 37, 51, and 84 days. Ages of the mice used as the odor donors varied between 3 and 6 months (adult but not old mice). The study was approved by the Indiana University Institutional Animal Care and Use Committee (IACUC). Food and water were provided *ad libitum*. Corn Cob bedding (#7097 Teklad Corn Cob Bedding, ¼.” Harlan Laboratories, Inc., Indianapolis) was used as bedding. The odor-receiver females were housed at 2 mice/cage to avoid the influence of isolation (Koyama, 1993a,b; Bartolomucci et al., 2009) and large group housing (Lee and van der Boot, 1955, 1956; Novotny et al., 1986; Ma et al., 1998; Koyama, 2004). The odor-donor mice were housed at 4 mice/cage. 2,5-Dimethylpyrazine, a chemical that induces Lee-Boot effect (Novotny et al., 1986; Ma et al., 1998; Koyama, 2004), was not detectable in the female mouse-bedding odor generated with this housing condition (see Odor Collection and Analysis for details on analyses of soiled bedding).

### Exposure to soiled bedding and synthetic pheromones

To expose mice to soiled bedding, mice were moved every 2 days for 7 days (4 times) to cages where odor-donor mice had been housed. Control mice were moved to clean cages. The bedding was used by odor-donor mice for 7 days. The period of exposure of 7 days was adapted from the studies of Mak et al. (2007) and Larsen et al. (2008). Those studies showed that both shorter (2 days) and longer (2 weeks) exposure periods did not induce enhanced neurogenesis. We also conducted a preliminary experiment exposing females to the bedding of odor donor males that were used for 2, 4, and 7 days, or to clean bedding. We determined that exposure to the bedding soiled by odor donor males for longer periods of time induced higher levels of cell proliferation in the SVZ of female mice (data not shown). We determined the concentrations of pheromones included in these soiled beddings using GC-MS (see Odor Collection and Analysis below) and found that the concentration of the farnesene isomers and SBT were increased in the bedding exposed to odor donors for longer periods (data not shown). These pheromones are normally volatile and odoriferous substances, however, their equilibrium concentrations in the air in the mouse cages is primarily controlled by forming complexes with the major urinary proteins (MUPs) (Zidek et al., 1999; Sharrow et al., 2002; Beynon and Hurst, 2003, 2004) that permit a slow release. The increased (measured) concentrations of pheromones in the headspace as a function of time are presumably due to (i) enhanced surface area in the bedding “coated” with urine; and (ii) perhaps to even larger degree, pheromone release from the MUPs due to the actions of microbial proteases [see Novotny (2003) for review]. Based on these preliminary experiments, the concentration of pheromone solutions for exposure of mice to synthetic pheromones was determined by the concentration of farnesene we detected in the soiled bedding used by odor donor mice for 7 days. For exposure to synthetic pheromones and benzothiazole, aqueous 250 ppm solutions (50 μ L) (or water for control) were directly deposited on the nostrils of mice (Jemiolo et al., 1986; Ma et al., 1999) twice daily for 7 days. The concentration of other pheromones was adjusted to the same concentration (250 ppm). This application frequency was based on the methods previously utilized, which successfully stimulated or suppressed estrus in females (Ma et al., 1999). The estrous status of the females was not controlled, but was evaluated at the time of perfusion. There were females of each estrous stage included in the samples but there was no obvious correlation between estrous status and proliferation of cells in the SVZ (data not shown).

### Marking and counting the proliferating cells

On the 8th day, mice were injected with 5-bromo-2-deoxyuridine (BrdU, B5002, Sigma-Aldrich) (300 mg/kgbw) 4 times every 2 h and intracardially perfused with paraformaldehyde 2 h after the last BrdU injection. Brains were post-fixed overnight at 4°C and stored in 15% sucrose in 0.2 M phosphate buffer until sectioning (16 μm, from Bregma distance 1.54 to −0.22 mm) using a cryostat (Leica CM1850, Leica Microsystems GmbH, Germany). Each section on slides was 80 μm apart. The sections were stored at −20°C until use, stained with anti-BrdU antibodies (Accurate Chemical Co., #OBT0030, 1:300, rat) and incubated with fluorescent secondary antibodies (Alexa Fluor 488, Invitrogen). Stained sections were imaged using Nikon Eclipse 80i (Nikon, Japan) at Indiana University METACyt core neuroscience labs for counting cells. BrdU^+^ cells were counted using Image-J software. The average of the number of BrdU^+^ cells on the left and right SVZ in 16 μm thick sections that were spaced 80 μm apart from Bregma distance 1.34 to −0.1, were analyzed. The length of the region along the ventricle was measured and the number of cells per millimeter was calculated, which was converted into the number of cells per unit length of SVZ at each Bregma distance. The total of these numbers were used as the data for each individual.

### Immunohistochemistry and measurement of fluorescence intensity

For observation of the expression of Sox2 in the proliferating cells at the SVZ, we conducted immunohistochemistry with anti-Sox2 (ab97959, rabbit, Abcam, Cambridge, MA) and anti-BrdU (Accurate Chemical Co., #OBT0030, 1:300, rat). Sodium citrate (0.01 M) was used for the denaturation process (Tang et al., 2007). Sections were washed with 0.1 M PBS and then treated with sodium citrate buffer for 20 min in a steamer (>99°C inside the steamer). After 20 min, the slides were incubated for 2 min in the sodium citrate buffer at room temperature to cool down. After the antigen recovery process, sections were washed with 0.1 M PBS before blocking with 10% normal goat serum in 0.1 M PBS for 1 h at room temperature. Then sections were kept at 4°C with the anti-BrdU and anti-Sox2 in 0.1 M PBS with 3% normal goat serum. On the second day, sections were washed with 0.1 M PBS, stained with secondary antibodies (goat anti-rat Alexa Fluor 488 and goat anti-rabbit Alexa Fluor594, 1:500, respectively, Invitrogen, Inc.) in 0.1 M PBS with 0.3% Triton X-100, and stained with Draq5 (#4084, 1:1000, Cell Signaling Technology, Beverly, MA). Stained sections were imaged using a Leica SP5 scanning confocal (Leica Microsystems GmbH, Germany) at The IUB Light Microscopy Imaging Center to generate the confocal images in Figures 3 and 6. Sox2^+^ cells were counted using Image-J software.

### Odor collection and analysis

Small magnetic stir bars provided with a polymer coating were used to absorb odor components. Stir bars (Twister™, 10 × 0.5 mm film thickness, 24 μL polydimethylsiloxane (PDMS) volume, Gerstel GmbH, Mülheim an der Ruhr, Germany) were also spiked with an internal standard (8 ng of 7-tridecanone) (Soini et al., 2006). Volatile and semi-volatile odor compounds were collected by passive diffusion (headspace mode) into the PDMS polymer by the sorption mechanism. To collect odors, the stir bars were placed in 0.5 mL polypropylene vials with drilled holes (0.5 mm, i.d.), placed in wire-mesh holders hung on the sides of the cages for up to 7 days.

The gas chromatograph-mass spectrometer (GC-MS) instrument was an Agilent 6890N gas chromatograph connected to a 5973i MSD mass spectrometer (Agilent Technologies, Inc., Wilmington, DE) with the Thermal Desorption Autosampler/Cooled Injection System (TDSA-CIS 4, Gerstel GmbH). A DB-5MS separation column (30 × 0.25 mm, i.d., 0.25 μm film thickness) capillary (Agilent, J&W Scientific, Folsom, CA) was employed.

### Test compounds

The farnesene mixture of isomers was obtained from Tokyo Kasei Kogyo Co., Ltd (Tokyo, Japan). Benzothiazole and 2,5-dimethylpyrazine were purchased from Sigma-Aldrich Chemical Company (Milwaukee, WI). Dehydro-*exo*-brevicomin (DHB) and SBT were synthesized in the Novotny laboratory and syntheses of them have been described in the literature (Wiesler et al., 1984; North and Pattenden, 1990). Test compounds were dissolved in OmniSolv® water (EM Science, Gibbstown, NJ).

### Statistics

Levene's test was used, confirming the homogeneity of variance in the data (*F* = 0.517, *df* = 7.4, *P* = 0.816), and the null hypothesis that all the variance are equal was accepted. ANOVA was used to compare the number of proliferating cells. For one-way ANOVA comparison with control conditions, Dunnett test was used for the *post-hoc* analyses. For two-way ANOVA comparisons with age differences and odor condition differences, Tukey's *post-hoc* analyses was used to determine the impact of pheromone at each age. For comparison of Sox2^+^ cells among the proliferation cells in the mice exposed to pheromone and control group, Mann-Whitney *U*-test was employed (Nachar, 2008). SYSTAT software was used for these statistical analyses. Curve fitting for the change of neurogenesis rate, depending on the concentration of pheromone solution, was conducted using MATLAB and R software.

## Results

Aqueous solutions of male murine pheromones, the farnesene isomers (α and β, 1:2 ratio), SBT, DHB, and female murine pheromone 2,5-DMPZ were evaluated for their ability to promote cell proliferation in the SVZ of female mice. The concentration (250 ppm) used was approximately equivalent to the amount of the farnesenes present in male-soiled bedding used by odor donor males for 1 week and this concentration was previously shown to induce estrus in female mice (Jemiolo et al., 1986; Ma et al., 1999). Benzothiazole was chosen as a control compound because it is ubiquitously present both in female and male mouse urine. No active roles in murine chemical signaling have been identified for urinary benzothiazole (Novotny, 2003; Novotny et al., 2007). The rate of SVZ cell proliferation after exposure to either male or female-soiled bedding (used by odor donors for 7 days) was assessed.

**Figure 1:**
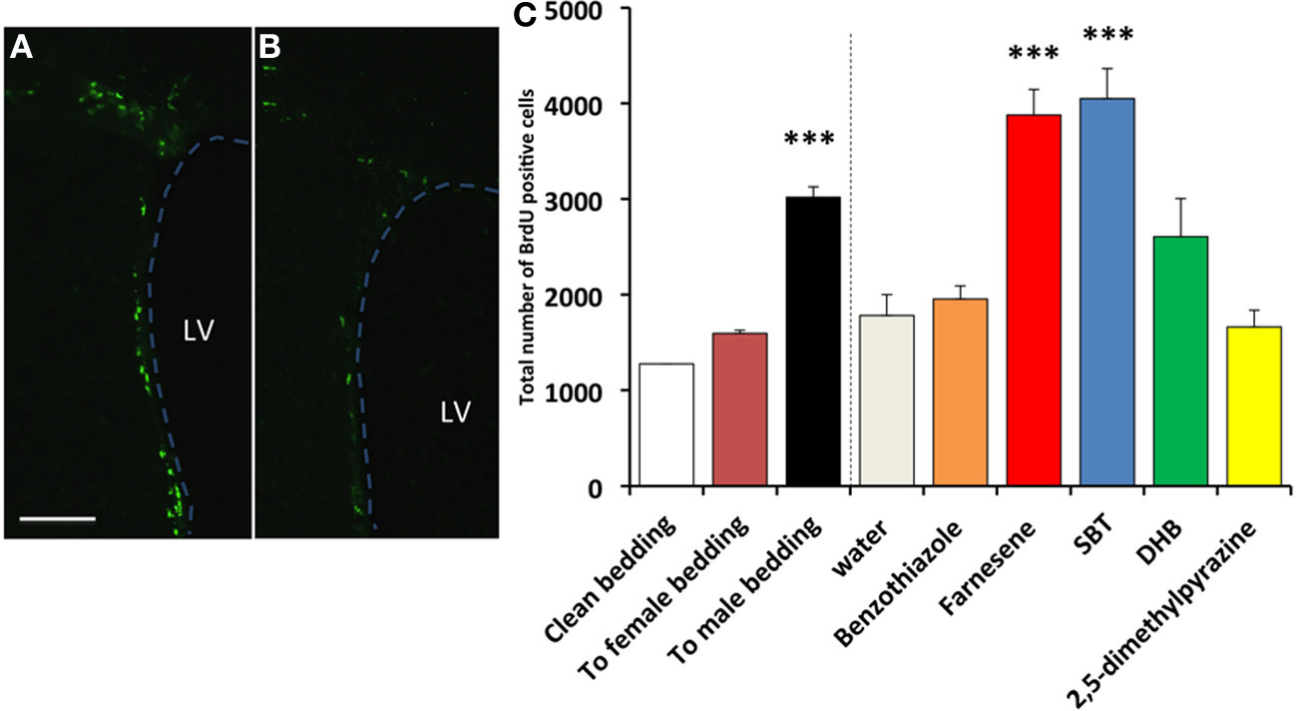
**Influence of exposure to soiled bedding and to synthetic analogs of mouse pheromones on the cell proliferation in the SVZ of female mice.** BrdU^+^ cells (green fluorescence) in the SVZ of females exposed to farnesene **(A)** or clean bedding **(B)**. LV: lateral ventricle. Dashed line indicates the border of lateral ventricle. Scale bar = 100 μm. **(C)** Total number of BrdU labeled cells (+SD) in the SVZ of female mouse. Female mice exposed to male-soiled bedding (*n* = 7) showed higher cell proliferation in the SVZ than the females exposed to clean bedding (*n* = 5) (*P* < 0.001). Female mice exposed to farnesene (*n* = 8) and SBT (“SBT,” *n* = 6) showed higher cell proliferation than females exposed to water (*n* = 4). Females exposed to dehydro-exo-brevicomin (“DHB”, *n* = 6), 2,5-dimethylpyrazine (*n* = 4), or benzothiazole (*n* = 5) did not show enhanced cell proliferation in the SVZ compared to females exposed to water. ^***^: significantly higher than control *P* < 0.001, Dunnett test.

In the SVZ of female mice, the number of BrdU^+^ cells in the SVZ of females exposed to male-soiled bedding (*n* = 7) was significantly higher than females exposed to clean bedding (*n* = 5), whereas females exposed to female-soiled bedding (*n* = 6) did not show a significant difference [*F*_(2, 15)_ = 33.759, *P* < 0.001; Dunnett test of *post-hoc* comparison with Clean bedding group, To male bedding group, *P* < 0.001, To female bedding group, *P* = 0.166] (Figure 1B left side of dashed line). The number of BrdU^+^ cells in the SVZ of females exposed to synthetic analogs of pheromones, the farnesenes (*n* = 8) and SBT (*n* = 6), was significantly higher than the females exposed to water (*n* = 4), whereas females exposed to DHB (*n* = 6), 2,5-DMPZ (*n* = 4), and benzothiazole (*n* = 5) did not show a significant difference [*F*_(5, 27)_ = 14.695, *P* < 0.001; Dunnett test of *post-hoc* comparison with Water group, Farnesene group and SBT group, *P* < 0.001 respectively, DHB group, *P* = 0.114, benzothiazole group, *P* = 0.498, 2,5-DMPZ, *P* = 0.332] (Figure 1A and right side of dashed line in Figure 1B). As the female murine pheromone 2,5-DMPZ suppresses estrus (Novotny et al., 1986; Ma et al., 1998; Koyama, 2004), we had hypothesized that it might suppress the rate of cell proliferation, however, this compound did not affect cell proliferation in females. It seems plausible that some of the pheromones from one sex may enhance the rate of cell proliferation in the SVZ of the opposite sex, with little or no effect on animals of the same sex.

**Figure 2:**
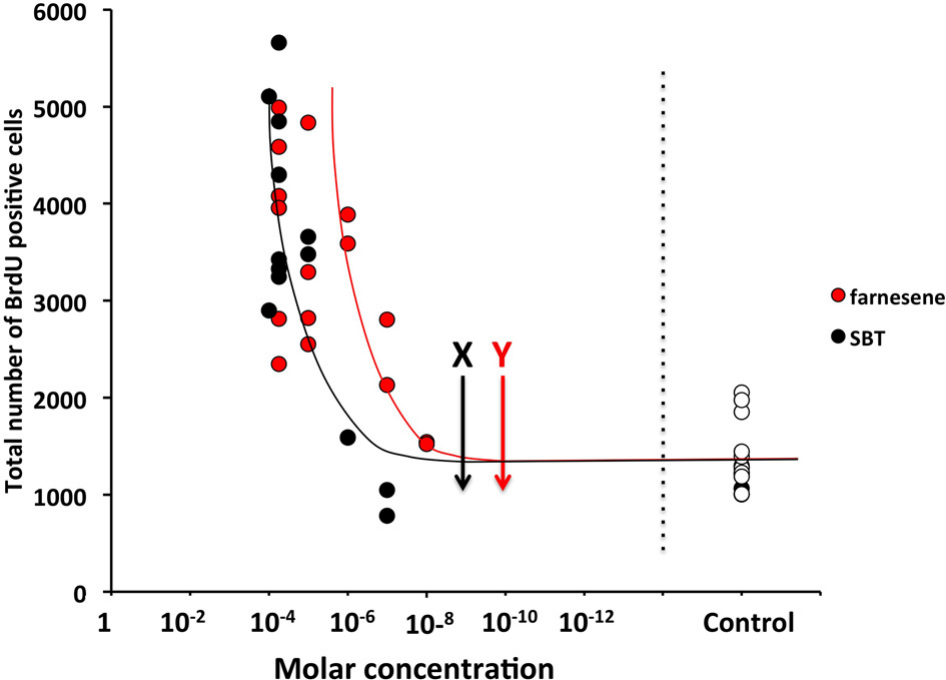
**Influence of various concentrations of male mouse pheromones, farnesenes and SBT, on cell proliferation in the SVZ of adult female mice.** Arrows with “X” and “Y” show the threshold concentrations of SBT and farnesenes at the vomeronasal neurons *in vitro* (Leinders-Zufall et al., 2000), respectively. Each dot indicates individual result and white dots show the results of controls.

The detection threshold of the farnesenes, SBT and DHB at the vomeronasal neuron is known to be as low as 10^−11^ to 10^−10^ M, 10^−10^ to 10^−9^ M, and 10^−10^ to 10^−9^ M respectively (Leinders-Zufall et al., 2000). In order to determine whether these levels also induced a detectable change in the rate of cell proliferation in the SVZ, we exposed females to a range of concentrations of the farnesenes and SBT, which had been shown to stimulate cell proliferation at 250 ppm (Figure 1). The rate of cell proliferation showed an exponential decay as the concentration decreased. We determined that the threshold concentration to induce enhanced cell proliferation was approximately 10^−6^ M for farnesene and 10^−5^ M for SBT (Figure 2). Although previous studies that showed the detection threshold at the vomeronasal neuron (Leinders-Zufall et al., 2000) utilized a slice preparation and our study used live animals, it is conceivable that a concentration higher than the detection threshold for the vomeronasal neurons is required to induce cell proliferation in the SVZ.

**Figure 3:**
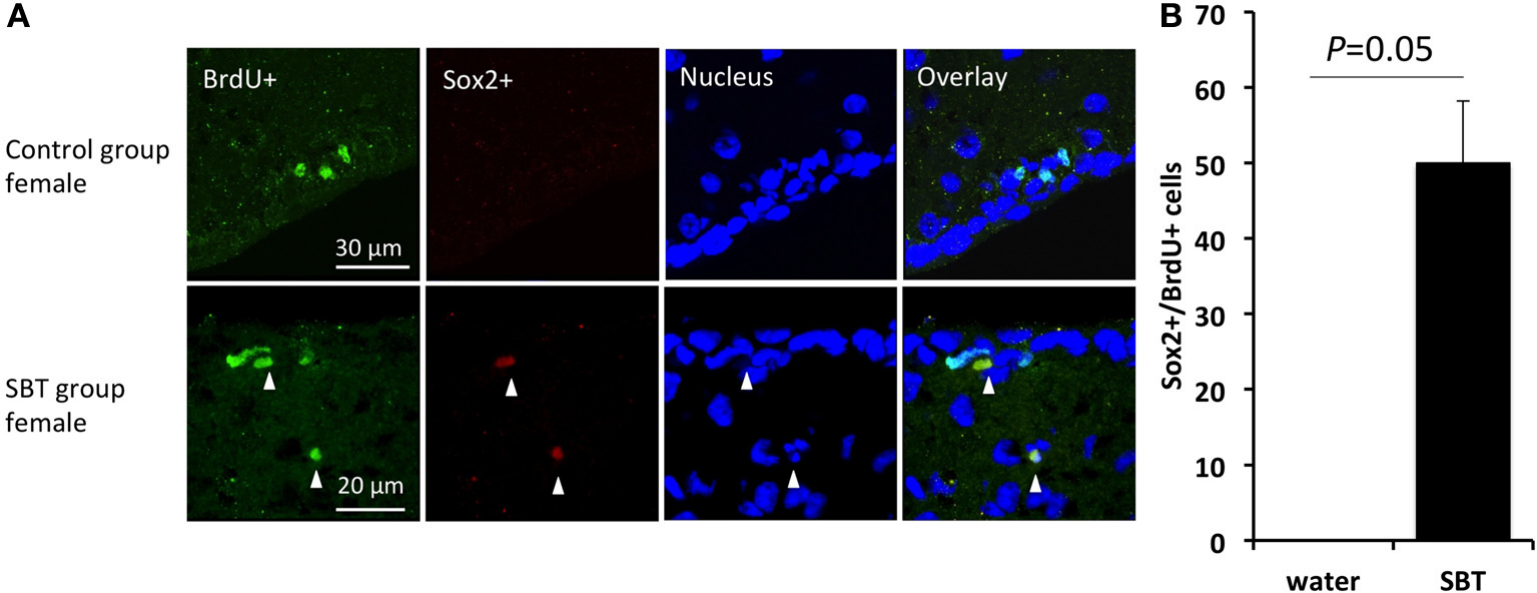
**Sox2 expression in the proliferating cells in the SVZ of female mice. (A)** Expression of Sox2 in the BrdU labeled cells (arrow heads). **(B)** BrdU labeled cells included more neuronal progenitors (Sox2^+^) in the females exposed to male mouse pheromone SBT (*n* = 3) than the control group females (*n* = 3). Bars indicate median and interquartile range.

Sox2, a HMG (high-mobility-group) box transcription factor gene, is expressed highly in adult neural progenitors and serves as one of the markers of neural stem/progenitor cells (Von Bohlen und Halbach, 2011). We used immunofluorescence to determine if exposure to the male synthetic pheromone SBT might enhance the generation of neural progenitor cells. SBT was selected for its strong influence on cell proliferation. We found that the number of Sox2^+^ cells in the proliferating cells in the SVZ was higher in the females exposed to SBT than in the control females (Mann-Whitney *U*-test, *U* = 0, *P* = 0.05) (Figure 3).

**Figure 4:**
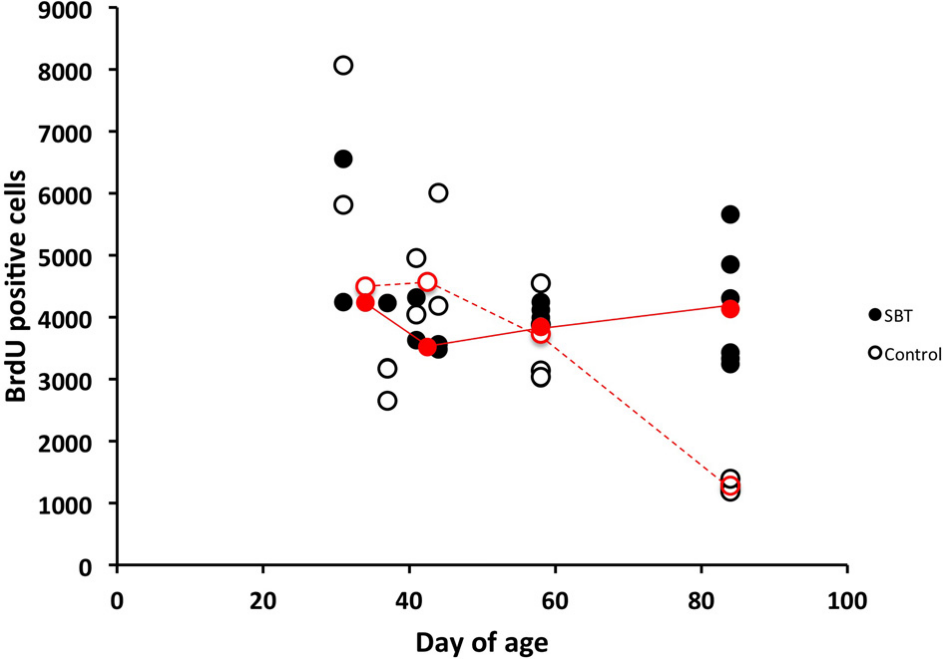
**Influence of SBT (250 ppm) on cell proliferation in the SVZ of females of various ages.** Male mouse pheromone stimulated cell proliferation in the SVZ of adult female mice but not in prepubertal-, or post-pubertal young female mice. Red circles indicate the medians at each age (filled red circles with solid lines indicate medians of females exposed to SBT and blank red circles with dashed lines indicate medians for control group females). Results of post-natal days 31 and 37 were used together to produce medians of post-natal day 31–37, and these of post-natal days 41 and 44 were used to produce medians of post-natal day 41–44.

We then measured the influence of male murine pheromones on cell proliferation in the SVZ of females at distinct developmental stages using SBT. We found that SBT enhanced cell proliferation in the SVZ of adult females (3-month-old) but not in the pre-pubertal (31-day-old, starting exposure to pheromone from 24-day-old), pubertal (37-day-old, starting exposure to pheromone from 30-day-old, which is the average age when the vagina opens) or young post-pubertal females (41-, 44- and 58-day-old; ages when exposure started were 7 days prior to these ages) (Figure 4). When the ages were grouped as being either near puberty (31- and 37-day old), as young post-pubertal females (41- and 44-day old), as young adults (58-day-old) or as adults (84-day-old), only the latter group showed a significant difference in cell proliferation in the SVZ between treated females and controls with the other groups not showing any significant differences [two-way ANOVA, age *F*_(3, 27)_ = 5.604, *P* = 0.004, odor condition *F*_(1, 27)_ = 1.093, *P* = 0.305, age*odor condition *F*_(3, 27)_ = 5.523, *P* = 0.004; Tukey's *post-hoc* test, age-matched pair-wise comparison of pheromone group vs. control group, near puberty, *n*_pheromone_ = 4, *n*_control_ = 4, *P* = 1; young post-pubertal, *n*_pheromone_ = 4, *n*_control_ = 4, *P* = 0.891, young adult, *n*_pheromone_ = 4, *n*_control_ = 4, *P* = 1, adult, *n*_pheromone_ = 64, *n*_control_ = 5, *P* = 0.006].

**Figure 5:**
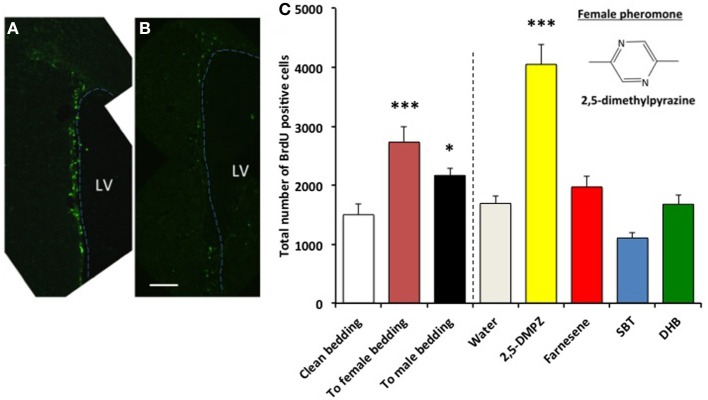
**Influence of exposure to female and male mouse pheromones on cell proliferation in the SVZ of male mice.** BrdU^+^ cells (green fluorescence) in the SVZ of males exposed to 2,5-DMPZ **(A)** or clean bedding **(B)**. LV: lateral ventricle. Dashed line indicates the border of lateral ventricle. Scale bar = 100 μm. **(C)** Male mice exposed to female mouse pheromone 2,5-DMPZ (*n* = 6) showed enhanced cell proliferation in the SVZ compared to males exposed to water (*n* = 7) (Dunnett test, *P* < 0.001). Male mice exposed to female-soiled bedding (*n* = 8) and male-soiled bedding (*n* = 7) showed higher cell proliferation in the SVZ compared to males exposed to clean bedding (*n* = 6). ^***^: *P* < 0.001, ^*^: *P* < 0.05. Chemical structure of 2,5-dimethylpyrazine is shown in the figure. Other pair-wise comparisons with control group were not significant.

**Figure 6:**
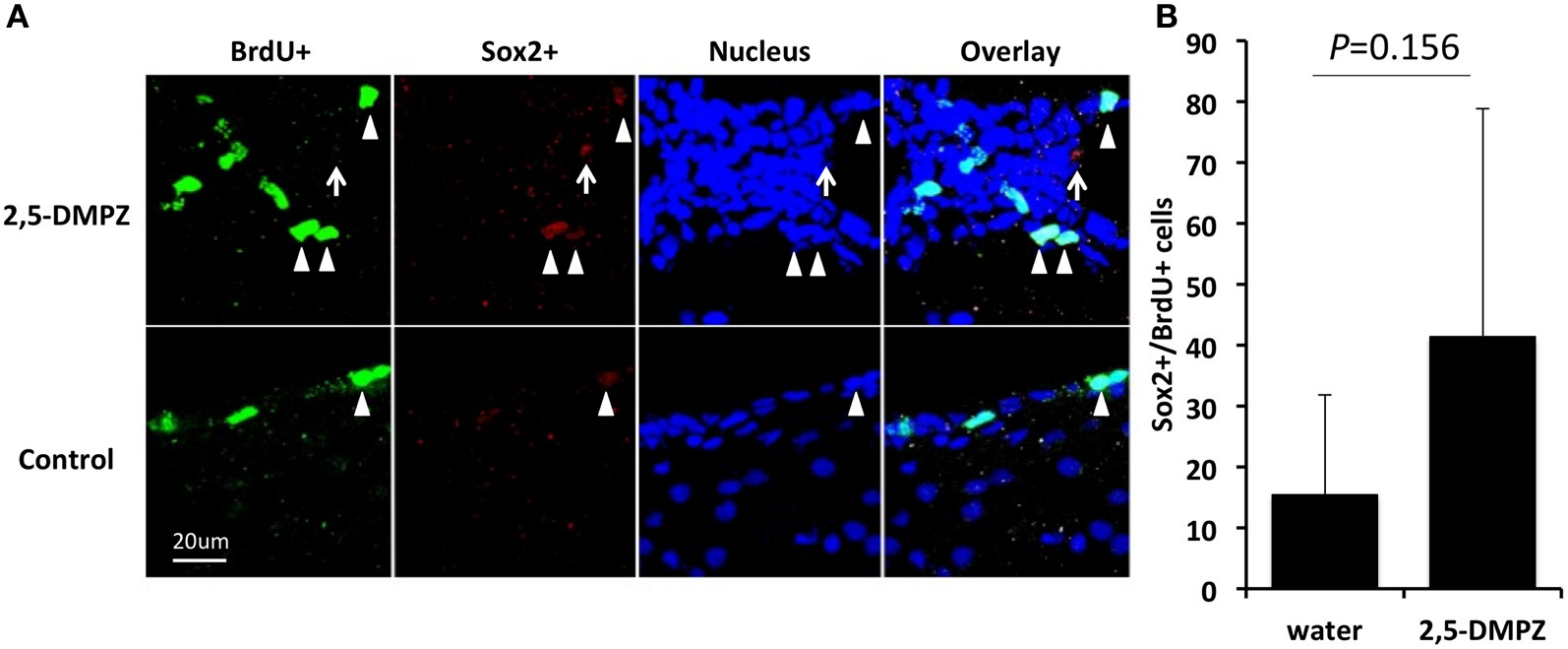
**Sox2 expression in the proliferating cells in the SVZ of male mice. (A)** Expression of Sox2 in the BrdU^+^ cells (arrow heads) in the SVZ of male mice exposed to 2,5-DMPZ (above) and control group male mice (below). **(B)** Rate of BrdU^+^ cells doubled labeled with Sox2^+^ in the males exposed to 2,5-DMPZ (*n* = 4) and control group males (*n* = 3). Sox2^+^ cells that did not overlap with BrdU^+^ cells and nucleus staining [arrow in **(A)**] were not counted. Bars indicate median and interquartile range.

Next, we tested whether a female murine pheromone (Novotny et al., 1986; Ma et al., 1998; Koyama, 2004) 2,5-DMPZ or male murine pheromones (SBT, the farnesene isomers, and DHB) might influence cell proliferation in the SVZ of males. We also exposed males to soiled bedding from either other males, from females or to clean bedding (control). We found that males exposed to either female-soiled bedding (*n* = 8) or male-soiled bedding (*n* = 7) showed significantly higher levels of cell proliferation in the SVZ compared to the males exposed to clean bedding (*n* = 6) [ANOVA, *F*_(2, 18)_ = 8.634; Dunnett *post-hoc* comparison with clean bedding group, To male bedding group *P* = 0.037, To female bedding group, *P* = 0.001] (Figure 5). The finding that the soiled bedding from males (the same sex) could stimulate cell proliferation in other males was distinctly different from the situation observed for females. Of the groups that were exposed to synthetic analogs of murine pheromones, only the group that was exposed to 2,5-DMPZ (*n* = 6) showed significantly higher cell proliferation in the SVZ whereas the male murine pheromones, the farnesenes, SBT, and DHB, did not stimulate or suppress cell proliferation in the SVZ of males [ANOVA, *F*_(4, 24)_ = 32.963, *P* < 0.001; Dunnett *post-hoc* comparison with Water group (*n* = 7), 2,5-DMPZ, *P* < 0.001, the farnesenes (*n* = 6), *P* = 0.353, SBT (*n* = 6), *P* = 0.063, DHB (*n* = 4), *P* = 0.5]. The number of BrdU^+^ cells in the SVZ of males exposed to the female pheromone 2,5-DMPZ (*n* = 4) that also stained with Sox2^+^ was not significantly different from that of control group males (*n* = 3) (Figures 6A,B) (Comparison of males exposed to 2,5-DMPZ and control group males, Mann-Whitney *U*-Test, *U* = 2.5, *P* = 0.156).

## Discussion

To our knowledge, these findings represent the first observation that single, synthetic pheromones promote cell proliferation in the SVZ in mammals. Previous studies using soiled bedding as a source of pheromones have reported their stimulatory impact on neurogenesis in females (Mak et al., 2007; Larsen et al., 2008; Oboti et al., 2009), however, in this report we are presenting the first evidence that female pheromones strongly promote cell proliferation in the SVZ of males.

We have demonstrated that previously recognized male murine pheromones, the farnesenes and SBT, are capable of enhancing cell proliferation in the SVZ of female mice. DHB did not significantly stimulate cell proliferation in the SVZ of female mice, which indicates that not all of the male murine pheromones tested were capable of stimulating cell proliferation in the female SVZ. In addition, we found that the female murine pheromone, 2,5-DMPZ (Novotny et al., 1986; Ma et al., 1998) was capable of enhancing cell proliferation in the male SVZ. Our findings suggest that both male and female pheromones, when presented as their synthetic analogs at concentrations detected in soiled bedding, are capable of stimulating cell proliferation in the brains of the opposite sex. We also found that these pheromones do not have detectable effects on brains of the same sex, i.e., the female murine pheromone 2,5-DMPZ did not affect cell proliferation in the SVZ of female mice and the male murine pheromones, the farnesenes, SBT, and DHB, did not affect cell proliferation in the SVZ of male mice. Previous studies have shown that pheromones have suppressive impact on the reproductive system within the same sex, for example, female pheromones suppressed estrus in females (Lee and van der Boot, 1955, 1956; Novotny et al., 1986; Ma et al., 1998) and dominant male odors suppressed sperm activity in subordinate males (Koyama and Kamimura, 1999, 2003). Our findings suggest that, on neurogenesis, pheromones do not have suppressive impact on the same sex. However, males exposed to male-soiled bedding showed an increased proliferation in the SVZ, although this was not observed in females exposed to female-soiled bedding. This suggests that for males, the ability of pheromones to promote neurogenesis may not only occur between the sexes. Further studies on the sex differences in the impact of pheromones on neurogenesis may be of importance.

The cells proliferating in the SVZ are known to be astrocyte-like cells expressing glial fibrillary acidic protein (GFAP) that are capable of giving rise to neuroblasts that migrate to the olfactory bulb and become interneurons (Whitman and Greer, 2009). Sox2 is a transcription factor that is involved in the proliferation of neural stem cells in the SVZ and subgranular zone (SGZ) and thus serves as one of the neural stem/progenitor cell markers (Bylund et al., 2003; Ferri et al., 2004; Ghashghaei et al., 2006; Suh et al., 2007; Favaro et al., 2009; Mu et al., 2010; Von Bohlen und Halbach, 2011; Yao et al., 2012). Sox2 was detected in a higher percentage of the proliferating cells in the SVZ of females exposed to male murine pheromones than in the males exposed to female pheromones. Although there is a possibility that it is due to the small sample size, these findings suggest that the effect of pheromone-induced cell proliferation, i.e., the cell fate, in males and females may also differ. Future studies will be required to determine the number and identity of the mature neuronal types that are generated.

As summarized above, cell proliferation/neurogenesis in the SVZ of females was strongly stimulated by male pheromones and cell proliferation in the SVZ of males was strongly stimulated by female pheromones. What are the potential molecular mechanisms that underly these effects? In our previous studies, we determined that sperm density was enhanced by exposure to female-soiled bedding (Koyama and Kamimura, 2000) and male pheromones stimulated females to come into estrus (Jemiolo et al., 1986; Ma et al., 1999; Koyama, 2004). As gonadal hormones have been previously shown to influence neurogenesis (Galea et al., 2006), it is possible that pheromones act via this class of effectors to stimulate neural cell proliferation. Prolactin has been shown to regulate neurogenesis in the SVZ of females in response to male-soiled bedding, and its infusion into males was also capable of stimulating neurogenesis (Mak and Weiss, 2010). In addition, estrogen was able to promote neurogenesis in the dentate gyrus (Mak et al., 2007). These studies suggest that some pheromones act via the sex hormones and that these in turn stimulate cell proliferation in the reproductive organs and in the brain. Previous studies have suggested that the increased neurogenesis in females induced by male-soiled bedding was capable of enhancing female preference for dominant males (Mak et al., 2007) and for fostering mate recognition (Oboti et al., 2011), which may be critical for avoiding pregnancy block (Bruce effect) (Bruce, 1959). The infusion of prolactin in males has been associated with an improved recognition of pups (Mak and Weiss, 2010) and the improved recognition of a male to its own pups may reduce infanticide, which happens with unrelated offspring (Vom Saal and Howard, 1982). The ability of pheromones to act via sex horomones to promote cell proliferation/neurogenesis is an area that merits further investigation.

The time required for newly born progenitor cells in the SVZ to be incorporated in the olfactory bulb as interneurons is about 3 weeks. Therefore, it is unlikely that biological effects that occur more rapidly would be mediated by the pheromonally-induced neurogenesis. 2,5-DMPZ is a female murine pheromone that is secreted by females when they are housed at higher density and it suppresses their estrus (Lee and van der Boot, 1955, 1956; Coppola and Vandenbergh, 1985; Novotny et al., 1986; Ma et al., 1998; Koyama, 2004). The male murine pheromones, SBT, the farnesenes, and DHB, will stimulate females to come into estrus and this will be mostly on the third day after exposure to male pheromones. The time interval from the start of regaining a short estrous cycle until delivery is ~22–26 days (3 days to come into estrus and 19 days of pregnancy lead to a total of 22 days and, if pregnancy starts at the next estrous day from some reason, it would be 26 days). This timeframe suggests that the newly incorporated interneurons could contribute to pup recognition, however, this interval is not consistent with a role in mate recognition, which takes place within 1 week of exposure. Thus, the enhanced neurogenesis may not be related to the Bruce effect, which is based on the memory of the mate, but may be involved in maternal/paternal recognition.

A previous study demonstrated that the bedding of dominant males increased the rate of neurogenesis in females, while that of subordinate males lacked this ability (Mak et al., 2007). Those investigators also demonstrated that females exposed to the bedding of dominant males showed a preference for dominant males in sociality tests, whereas females exposed to the bedding of subordinate males did not show any preference to either dominant or subordinate males. Dominant males secrete the murine pheromones, the farnesenes, SBT and DHB, at higher levels than subordinate males (Novotny et al., 1990). Together, these results suggest that the farnesenes and SBT could serve as the pheromones involved in establishing females' preference for dominant males by stimulating their neurogenesis. Studies on the biology of house mice have shown that these mice establish social groups with a despotic type of social dominance (Uhrich, 1938) and a dominant male sires most of the offspring (Berry and Bronson, 1992). It is beneficial for female mice to be able to identify males of higher social status and to have a preference for these males to ensure their reproductive success.

In addition to the impact on males' and females' reproductive conditions and neural cell proliferation, these male murine pheromones are known to stimulate aggressive behaviors in other males as well (Novotny et al., 1985; Koyama, 2004; Rodriguez and Boehm, 2009; Tirindelli et al., 2009). It might be that these pheromones stimulate the neural circuit that stimulates GnRH-expressing neurons (Boehm et al., 2005) in both females and males. The stimulation of GnRH neurons may, in females, increase the secretion of estrogen and induce them to come into estrus, and, in males, increase the secretion of testosterone, which promotes aggressive behaviors (Bronson and Desjardin, 1968; Davidson and Levine, 1972). Therefore, the same pheromone molecules can produce multiple responses in the same neural circuits.

Previous studies have shown that exposure of females to male-soiled bedding has enhanced the integration of newborn neurons (Oboti et al., 2009). Integration of newborn neurons is known to be increased by various factors including exercise (running), an enriched environment (van Praag et al., 1999a,b) and mating (Portillo et al., 2012). One of our future goals will be to determine if the exposure to synthetic analogs of pheromones enhances the integration of neurons in males and females. The elucidation of the mechanisms by which specific compounds influence cell proliferation and neurogenesis, and eventually reproductive conditions in males and females should provide further insight as to how the odor environment controls and modulates the reproductive activities of animals. The use of individual murine pheromones in such studies should permit an improved understanding of the neural circuitry underlying this process.

### Conflict of interest statement

The authors declare that the research was conducted in the absence of any commercial or financial relationships that could be construed as a potential conflict of interest.
